# Novel Bioactive Peptides from *Meretrix meretrix* Protect *Caenorhabditis elegans* against Free Radical-Induced Oxidative Stress through the Stress Response Factor DAF-16/FOXO

**DOI:** 10.3390/md16110444

**Published:** 2018-11-11

**Authors:** Weizhang Jia, Qiong Peng, Linnan Su, Xuesong Yu, Chung Wah Ma, Ming Liang, Xiquan Yin, Yongdong Zou, Zebo Huang

**Affiliations:** 1 Center for Bioresources and Drug Discovery, School of Biosciences and Biopharmaceutics, Guangdong Pharmaceutical University, Guangzhou 510006, China; jiawzh@gdpu.edu.cn (W.J.); pengqbetter@163.com (Q.P.); sulinnan1234@foxmail.com (L.S.); axuesong@163.com (X.Y.); 2 Research and Development Center, Infinitus (China) Company Ltd., Guangzhou 510665, China; william.ma@infinitus-int.com (C.W.M.); fiona.liang@infinitus-int.com (M.L.); xiquan.yin@infinitus-int.com (X.Y.); 3 Shenzhen Key Laboratory of Microbiology and Gene Engineering, College of Life Sciences and Oceanography, Shenzhen University, Shenzhen 518060, China; zouyd@szu.edu.cn; 4 School of Food Science and Engineering, South China University of Technology, Guangzhou 510641, China

**Keywords:** antioxidant peptides, oxidative stress, reactive oxygen species, transcription factor DAF-16/FOXO, *Meretrix meretrix*, *Caenorhabditis elegans*

## Abstract

The hard clam *Meretrix meretrix*, which has been traditionally used as medicine and seafood, was used in this study to isolate antioxidant peptides. First, a peptide-rich extract was tested for its protective effect against paraquat-induced oxidative stress using the nematode model *Caenorhabditis elegans*. Then, three novel antioxidant peptides; MmP4 (LSDRLEETGGASS), MmP11 (KEGCREPETEKGHR) and MmP19 (IVTNWDDMEK), were identified and were found to increase the resistance of nematodes against paraquat. Circular dichroism spectroscopy revealed that MmP4 was predominantly in beta-sheet conformation, while MmP11 and MmP19 were primarily in random coil conformation. Using transgenic nematode models, the peptides were shown to promote nuclear translocation of the DAF-16/FOXO transcription factor, a pivotal regulator of stress response and lifespan, and induce the expression of superoxide dismutase 3 (SOD-3), an antioxidant enzyme. Analysis of DAF-16 target genes by real-time PCR reveals that *sod-3* was up-regulated by MmP4, MmP11 and MmP19 while *ctl-1* and *ctl-2* were also up-regulated by MmP4. Further examination of *daf-16* using RNA interference suggests that the peptide-increased resistance of *C. elegans* to oxidative stress was DAF-16 dependent. Taken together, these data demonstrate the antioxidant activity of *M. meretrix* peptides, which are associated with activation of the stress response factor DAF-16 and regulation of the antioxidant enzyme genes.

## 1. Introduction

In aging, oxidative stress occurs when the equilibrium between oxidant and antioxidant is disturbed, tipping the redox balance toward an oxidative status. This often causes oxidative modification of cellular constituents such as proteins, nucleotides and lipids, leading to the disruption of cellular metabolism and signaling pathways [1,2]. It has been well established that the accumulation of oxidized biomolecules and associated cell damages are implicated in the pathogenesis of many diseases, including metabolic, cardiovascular and neurodegenerative diseases, most of which are age-related [3,4]. Therefore, protecting normal cells from harmful assaults of either endogenous or exogenous oxidants is an important direction for the prevention and treatment of age-associated diseases.

Reactive oxygen species (ROS) are continually generated as by-products in a number of cellular processes and are also produced when cells are exposed to a variety of external stimuli, such as ultraviolet light, ionizing radiation and other environmental stresses [5,6]. While a homeostatic level of ROS is necessary for normal functions in cells, including intracellular signaling and cellular communication [7], excessive ROS can cause detrimental damage. When an oxidative stress is sensed, the endogenous antioxidants of cells provide a first line of defense against the potentially deleterious effects by reducing the levels of the oxidants. Among the key endogenous antioxidants are superoxide dismutases (SOD), catalases (CAT) and glutathione S-transferases (GST), which work closely to protect cells from ROS-mediated damage [8]. Although cells and organisms can respond to stress signals continuously and attempt to maintain normal physiological balance, it may be necessary in the face of persistent and severe stress to readjust the signal network by exogenous bioactives to restore the homeostasis, otherwise diseases may occur [9].

Bioactive natural products, including peptides, polysaccharides and polyphenols, are increasingly drawing attention to their physiological and pharmacological activities. For example, natural antioxidants are shown to help alleviate oxidative stress, delay aging and alleviate the progression of age-related diseases [10,11,12,13,14,15,16]. Antioxidant peptides, for instance, have been obtained from a variety of natural sources and used as bioactive ingredients, and their bioactivities as well as mechanisms of action are studied in both in vitro and in vivo systems [16,17,18,19]. The Asiatic hard clam *Meretrix meretrix* Linnaeus is an economically important bivalve clam species in China, where it has been used as a traditional medicine and also as an enticing seafood. According to *Essential Prescriptions from the Golden Cabinet* (Jin-Gui-Yao-Lue), a classic traditional Chinese medicine book by Zhang Zhongjing (150–219) of the Eastern Han dynasty, *M. meretrix* was used to treat polydipsia, which is a condition of excessive thirst and one of the initial symptoms of diabetes in modern medicine. In *Compendium of Materia Medica* (Ben-Cao-Gang-Mu) compiled by Li Shizhen (1518–1593) in the Ming dynasty, it was used to treat furuncle and detoxify alcohol. Recent studies have shown that *M. meretrix* extracts have a range of biological activities, including antioxidant, antimicrobial, anti-inflammatory and antitumor properties [20,21,22,23]. In particular, a peptide-rich extract of *M. meretrix* was found to have antioxidant activity in non-alcoholic fatty liver disease mice by dietary supplementation [24,25]. Nevertheless, sequence information and regulation mechanisms of bioactive peptides in *M. meretrix* are lacking. Here we first examined the in vivo antioxidant properties of a peptide-rich extract of *M. meretrix* using *Caenorhabditis elegans*, a powerful model animal widely used in biomedical studies [26]. After separation of the antioxidant peptide fractions, we then identified the peptide sequences and synthesized the peptides for bioactivity studies. Using the synthetic counterparts, we further investigated the bioactivities and mechanisms of action of the identified *M. meretrix* peptides, including the activation of the transcription factor DAF-16/FOXO and the regulation of stress response genes.

## 2. Results

### 2.1. Separation of Antioxidant Peptide Fractions from M. meretrix Peptide-Rich Extract

**Figure 1:**
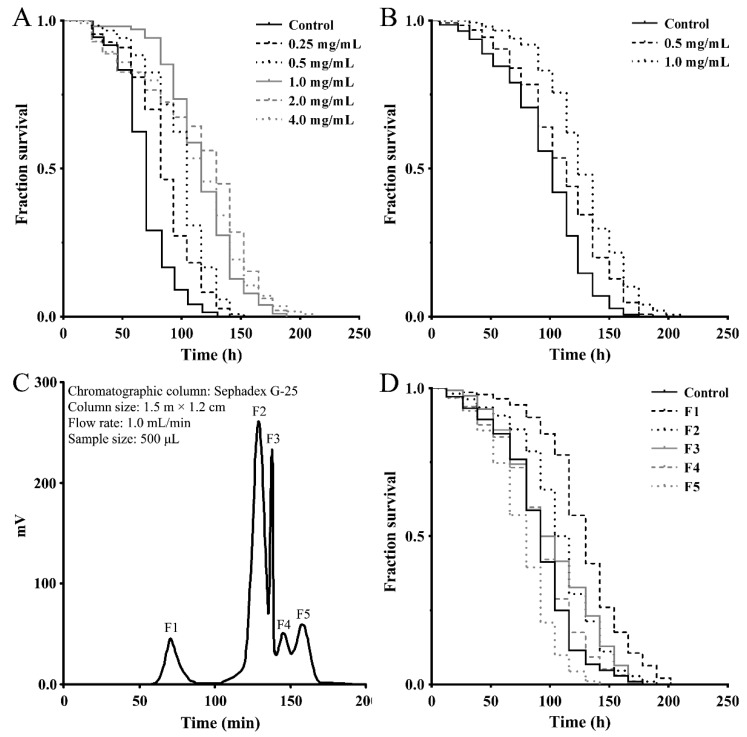
Antioxidant activities of the peptide-rich extract and the separeted peptide fractions from *M. meretrix*. (**A**) Paraquat resistance assay of the peptide-rich extract. (**B**) Paraquat resistance assay of the <3 kDa peptide fraction. (**C**) Gel chromatography separation of the <3 kDa peptide fraction by Sephadex G-25. (**D**) Paraquat resistance assay of the F1, F2, F3, F4 and F5 subfractions (1 mg/mL) from (C). The young adult nematodes were treated with the samples at indicated concentrations for 24 h prior to exposure to 50 mM of paraquat. Survival rates were scored every 12 h and data are shown as representative Kaplan-Meier survival curves.

**Figure 2:**
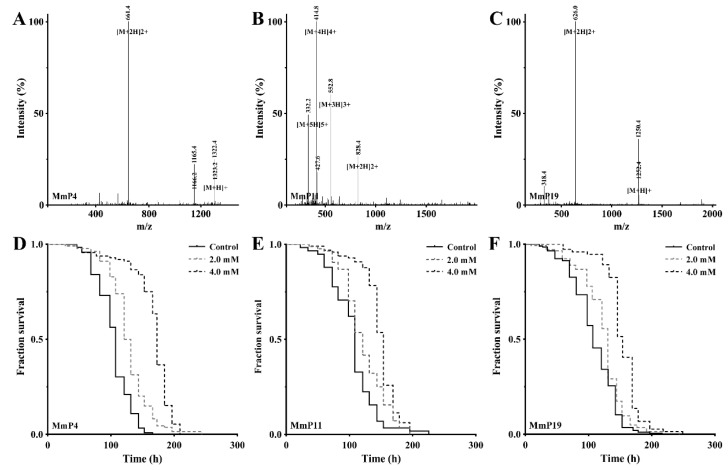
Mass spectra and antioxidant activities of synthetic peptides originated from *M. meretrix* peptides. The mass spectra of MmP4 (LSDRLEETGGASS), MmP11 (KEGCREPETEKGHR and MmP19 (IVTNWDDMEK) were shown in (**A**–**C**), respectively. The paraquat resistance assay was performed as in Figure 1, and representative Kaplan-Meier survival curves were shown for the nematodes treated with the peptides (**D**) MmP4, (**E**) MmP11 and (**F**) MmP19 at indicated concentrations.

To assess the effect of a *M. meretrix* peptide-rich extract on stress-related response, we first examined its antioxidant activity using a paraquat resistance assay in wild-type *C. elegans*. As shown in Figure 1A, the survival rate of the nematodes that were pretreated with the indicated concentrations of extract prior to paraquat exposure (50 mM) was consistently higher than that of the control nematodes exposed to paraquat alone, demonstrating the antioxidant capacity of the peptide-rich extract. To obtain stress-resistant peptide fractions, the *M. meretrix* peptide-rich extract was separated into <3 kDa and >3 kDa fractions using ultrafiltration tubes with a 3 kDa molecular weight cut off (MWCO) and both fractions were subjected to an antioxidant test as above using the paraquat resistance assay. As shown in Figure 1B, the <3 kDa peptide fraction has better antioxidant activity and can significantly increase the survival rate of nematodes (>3 kDa not shown). The peptide fraction (<3 kDa) was then further separated by gel-filtration chromatography on a Sephadex G-25 column, and five subfractions (F1–F5) were obtained (Figure 1C). These subfractions were tested for their antioxidant ability as above using the paraquat resistance assay in the nematodes. As shown in Figure 2D, pretreatment with 1.0 mg/mL of the subfractions F1, F2 and F3 was able to increase the survival rate of the paraquat-exposed nematodes, demonstrating the antioxidant capacity of these subfractions. As subfraction F1 showed a better effect, this fraction was used for further experiments.

### 2.2. Identification, Synthesis and Characterization of Antioxidant M. meretrix Peptides

The peptide components of subfraction F1 were identified by RP-nano-LC-MS/MS analysis coupled with database-assisted peptide sequencing. In total, 25 peptides were found, which were named MmP followed by a number (Table 1). To determine which peptides contributed to the general antioxidant activity of subfraction F1, all 25 peptides were synthesized individually with a purity of >95% (Table 1) and used for antioxidant screening as shown above in the nematodes. The effective concentrations of the synthetic peptides were shown to be >1.0 mM as tested preliminarily and thus all the peptides were subsequently screened for antioxidant activity at 2.0 and 4.0 mM. As shown in Figure 2, the *M. meretrix* peptides MmP4, MmP11 and MmP19 were found to increase the survival rate of the nematodes after paraquat exposure, demonstrating the antioxidant activity of these peptides.

**Figure 3:**
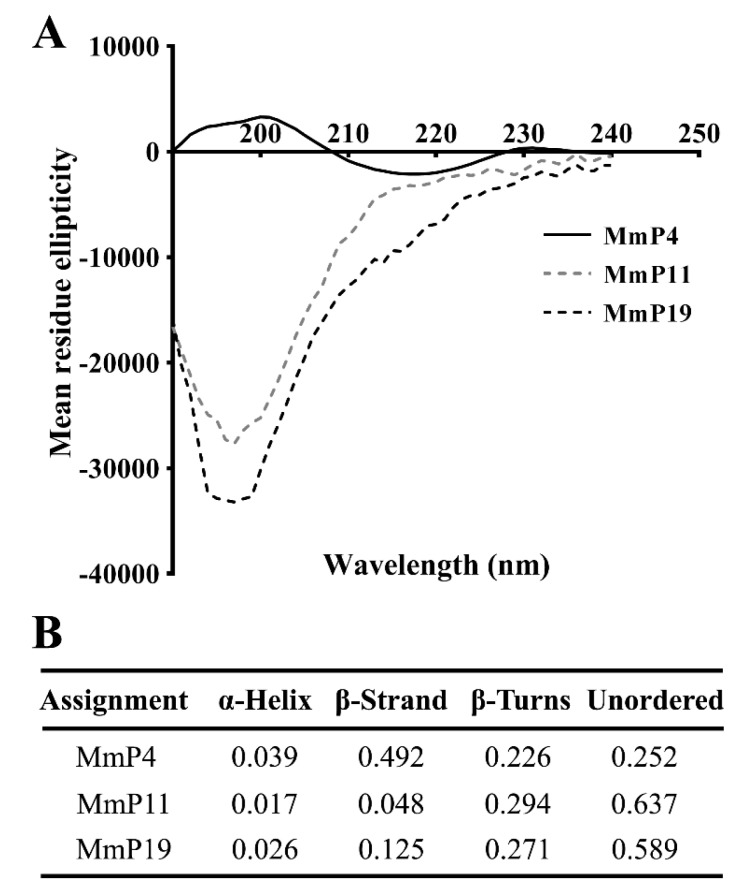
CD spectra and proportions of secondary structures of antioxidant *M. meretrix* peptides. CD spectra of synthetic peptides (**A**) were determined in deionized water at a concentration of 0.15 mg/mL, and the proportions of secondary structures (**B**) were calculated from the spectra.

To characterize the structural features of the antioxidant peptides, individual peptides in deionized water were analyzed by circular dichroism (CD) spectroscopy, which is a primary tool for rapid determination of the secondary structure and folding properties of proteins [27,28]. As shown in Figure 3, the CD spectra of MmP4 showed a positive peak at around 200 nm and a negative peak at around 218 nm, which is similar to that generally found for the β-sheet secondary structures [28,29]. Interestingly, the CD spectra of MmP11 and MmP19 showed a strong negative peak at around 195 nm, indicating a random coil in the peptides [28,29]. These data suggest that peptides with different secondary structures may have similar antioxidant activities.

### 2.3. Effects of Antioxidant M. meretrix Peptides on Subcellular Localization of the Transcription Factor DAF-16

**Figure 4:**
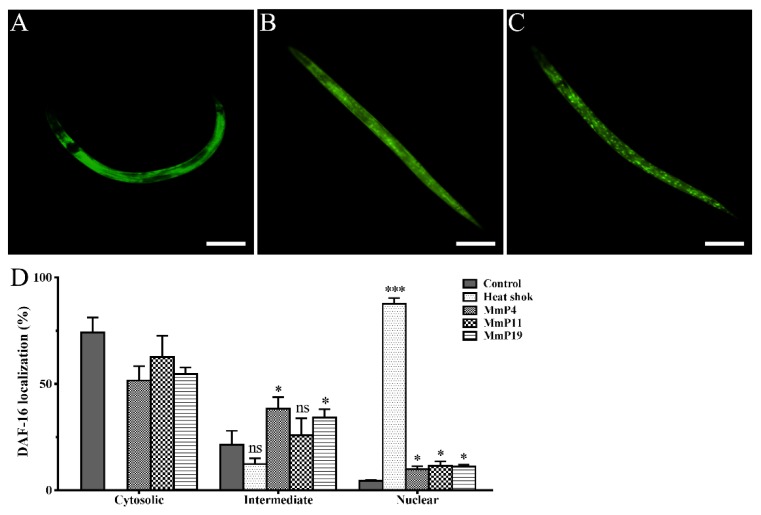
Effect of antioxidant *M. meretrix* peptides on cellular localization of the transcription factor DAF-16 in *C. elegans*. Synchronized L1 stage GR1352 nematodes were incubated for 24 h at 20 °C and treated with the peptides for 24 h; a portion of the adult nematodes without peptide treatment were heat-shocked at 35 °C for 1 h and used as a positive control. Cytosolic (**A**), intermediate (**B**) and nuclear (**C**) categories of DAF-16 localization were scored in approximately 30 animals per condition, and the data are presented as percentages of subcellular categories (**D**). Three independent biological replicates were performed. * *P* < 0.05; ** *P* < 0.01; *** *P* < 0.001. Scale bar: 200 μm.

DAF-16 is a *C. elegans* orthologue to the mammalian FOXO proteins, which play important roles in stress resistance and longevity [30,31,32]. Therefore, we used the transgenic *C. elegans* strain GR1352, which has been previously used to quantify DAF-16 nuclear localization and monitor relevant signaling activity [33,34], in order to determine the effects of *M. meretrix* peptides on DAF-16::GFP localization. Synchronized GR1352 larvae were first incubated at 20 °C for 24 h and then treated with or without the peptides at 20 °C for 24 h, while a portion of the adult nematodes without peptide treatment were heat-shocked at 35 °C for 1 h and used as a positive control of DAF-16 nuclear translocation. As shown in Figure 4, the majority of the control nematodes (~75%), which were incubated at normal conditions without peptide treatment, showed a cytosolic DAF-16::GFP localization with only <5% of the nematodes displaying a nuclear DAF-16::GFP localization. On the other hand, the majority of the heat-shocked nematodes (>85%) showed a nuclear DAF-16::GFP localization. Interestingly, when the nematodes were treated with 4.0 mM of *M. meretrix* peptides at normal conditions, the percentage of nematodes with detectable nuclear DAF-16::GFP localization was noticeably increased as compared to the control, indicating a partial translocation of DAF-16 from cytoplasm to nuclei by the peptides.

### 2.4. Effects of Antioxidant M. meretrix Peptides on the Expression of Oxidative Stress Related Genes

**Figure 5:**
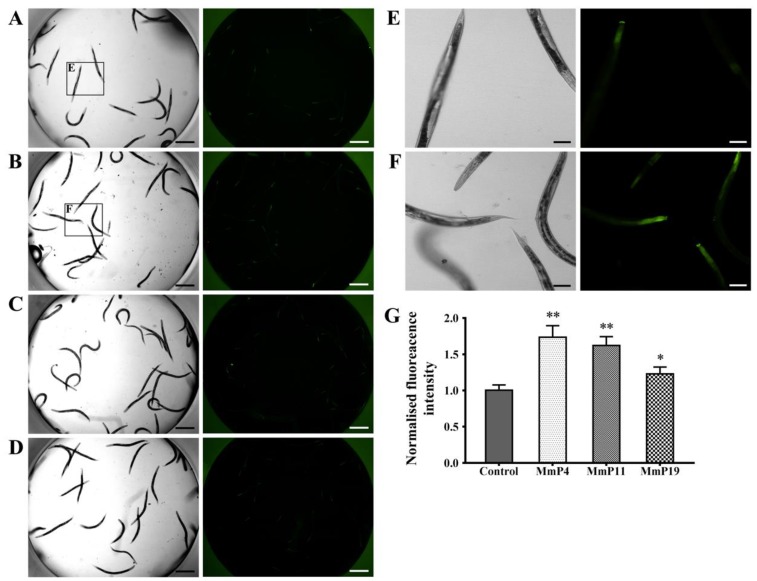
Effect of antioxidant *M. meretrix* peptides on the expression of SOD-3 in *C. elegans*. Synchronized CF1553 nematodes expressing the SOD-3::GFP reporter were incubated for 24 h at 20 °C and then treated with 4.0 mM of the peptides for another 24 h. The nematodes were collected for fluorescence detection of the control (**A**), MmP4 (**B**), MmP11 (**C**) and MmP19 (**D**). Scale bar: 0.5 mm. Image magnifications of the control (A) and MmP4 treatment (B) were also presented in (**E**) and (**F**), respectively. Scale bar: 0.1 mm. SOD-3::GFP fluorescence intensity was calculated from the images and shown in (**G**). Columns represent pooled normalized values of three independent biological replicates. * *P* < 0.05; ** *P* < 0.01.

**Figure 6:**
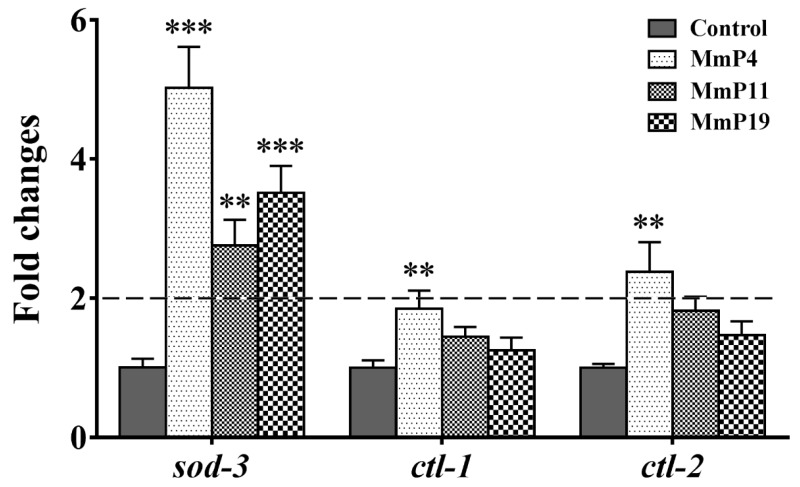
Effect of antioxidant *M. meretrix* peptides on the expression of *sod-3*, *ctl-1* and *ctl-2* in wild-type *C. elegans*. Synchronized N2 nematode larvae were incubated for 24 h at 20 °C and then treated with the peptides for 24 h. The expression of *sod-3*, *ctl-1* and *ctl-2* was analyzed by real-time PCR with three independent biological replicates. ** *P* < 0.01; *** *P* < 0.001.

It is known that DAF-16 plays a critical role in regulating the transcription of several antioxidant genes [35]. Since *M. meretrix* peptides were shown as above to have antioxidant and anti-aging activities, we used the transgenic strain *C. elegans* CF1553, which expresses a SOD-3::GFP reporter, to detect the effects of the peptides on SOD-3 expression [36]. After treatment with the peptides for 24 h, the SOD-3::GFP fluorescence level in the nematodes treated with MmP4, MmP11 and MmP19 was increased as compared to the control, suggesting the expression of the antioxidant enzyme SOD-3 was up-regulated by these peptides (Figure 5). To further investigate the effects of *M. meretrix* peptides on oxidative stress-related genes, we determined the expression of the antioxidant enzyme genes *sod-3*, *ctl-1* and *ctl-2* in wild-type *C. elegans* by real-time PCR. As shown in Figure 6, the *sod-3* transcript level was increased (>2 folds) in nematodes treated with 4.0 mM of MmP4, MmP11 and MmP19, while the *ctl-1* and *ctl-2* transcript levels were also increased (~2 folds) by the peptide MmP4.

### 2.5. Dependency of Antioxidant Activity of M. meretrix Peptides on Transcription Factor DAF-16

**Figure 7:**
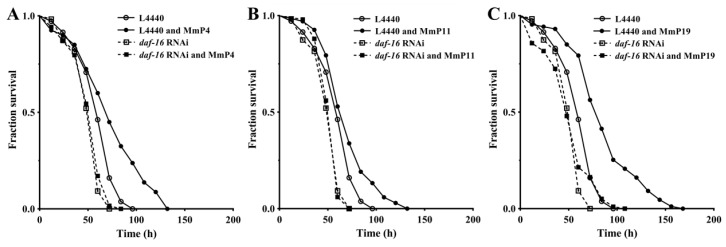
Antioxidant activity of *M. meretrix* peptides in wild-type *C. elegans* after *daf-16* RNA interface (RNAi). The nematodes with or without *daf-16* suppression by RNAi were treated with 4.0 mM of the peptides MmP4 (**A**), MmP11 (**B**) and MmP19 (**C**) and then subjected to paraquat resistance assay as in Figure 1.

As shown above, activation of the transcription factor DAF-16 may have an important role in promoting oxidative stress resistance by the *M. meretrix* peptides, as well as in regulating antioxidant enzyme genes. Therefore, we further determined the antioxidant activity of *M. meretrix* peptides in *C. elegans* after *daf-16* RNA interference (RNAi) to assess whether their antioxidant capacity was DAF-16-dependent. As shown in Figure 7, the oxidative stress resistance of *C. elegans* with the vector L4440 and without *daf-16* RNAi was significantly increased by the tested *M. meretrix* peptides (“L4440” vs. “L4440 and peptides”), but the increase was abolished under *daf-16* RNAi conditions (“*daf-16* RNAi” vs. “*daf-16* RNAi and peptides”), demonstrating that the increased resistance of the nematodes to oxidative stress by *M. meretrix* peptides was dependent on the transcriptional regulator DAF-16.

## 3. Discussion

ROS generated from both endogenous and exogenous sources is increasingly recognized as a pivotal mediator of many stress responses, and an imbalance between ROS production and elimination is considered a risk factor for a number of age-related diseases [37]. Peptides derived from a variety of food sources have shown antioxidant, anti-inflammatory, antitumor and other bioactive properties and thus may have beneficial potential in promoting health and preventing diseases [38]. Several peptides isolated from *M. meretrix*, a hard clam with high nutritional and medicinal values, have recently been shown to have apoptosis-inducing and antitumor activities [20,21,22]. Using the animal model *C. elegans*, we demonstrate in this study the in vivo antioxidant activities of a *M. meretrix* peptide extract and its peptide components, which were identified by RP-nano-LC-MS/MS and individually synthesized for bioactivity studies. We then reveal that the antioxidant *M. meretrix* peptides were able to regulate the expression of oxidative stress-related genes and that their antioxidant activity was dependent on the transcription factor DAF-16/FOXO, an evolutionarily conserved stress response and lifespan regulator [39,40].

The biological activities of a protein are, in theory, the functional manifestations of the amino acids it contains, and some short peptide segments may have specific roles in the overall function of the protein but not all segments are equally important [41]. It is noteworthy that the amino or carboxyl terminals of some of the identified *M. meretrix* peptides in this study, e.g., MmP4, are branched-chain amino acids (leucine, isoleucine and valine), which may have potential antioxidant activities [42]. CD spectroscopy is a valuable method for the analysis of peptide secondary structures, including α-helix, β-sheet, β-turn and random coil [27,28]. CD spectra of the antioxidant *M. meretrix* peptides revealed that MmP4 was able to form a secondary structure of β-sheet, but MmP11 and MmP19 were primarily in random coil conformation, suggesting that the antioxidant activity peptides are not necessarily related to a specific secondary structure and that more detailed structure-activity studies of peptides are needed.

It is established that high concentrations of paraquat can cause significant oxidative stress and trigger the occurrence of a pathological state [43,44]. In this study, we show that treatment with *M. meretrix* peptides significantly increased the resistance of *C. elegans* to paraquat-induced oxidative stress. Interestingly, other antioxidants such as C-phycocyanin and vitamin C can also be used in treatment against paraquat injuries [45,46]. The main mechanisms involved in the protective effects of these antioxidants include the reduction of both oxidative stress and inflammation, and induction of antioxidant defenses [44]. In agreement with this, the present study demonstrates that the antioxidant *M. meretrix* peptides were capable of up-regulating the expression of the antioxidant enzyme SOD-3. Further studies using *daf-16* RNAi reveal that the enhanced resistance to oxidative stress by the peptides was dependent on the transcriptional regulator DAF-16.

The transcription factor DAF-16 has been shown to play a variety of roles in regulating biological processes, including stress response and longevity [30,31,32]. For example, the exposure of nematodes to low concentrations of paraquat causes nuclear localization of DAF-16 and increases the expression of the DAF-16 transcriptional target gene *sod-3* [47]. Interestingly, the *daf-16* mutant is more sensitive to paraquat and heat stresses as compared to wild-type nematodes [48]. In the present study, the antioxidant *M. meretrix* peptides were shown to activate DAF-16 and increase expression of the antioxidant enzyme genes *sod-3, ctl-1* and *ctl-2* in different degrees. A number of studies have shown that decreased expression of *sod-3, ctl-1* or *ctl-2* can shorten the lifespan of *daf-2* nematodes, suggesting that these genes may contribute to the longevity of *daf-2* mutants [8,31,32,33,34,35]. Therefore, these results suggest that the antioxidant *M. meretrix* peptides may have dietary health benefits, including stress resistance, disease prevention and aging delay. Future work is needed to elucidate the absorption and distribution patterns of the peptides in nematodes after feeding and to investigate the effectiveness of the peptides in mouse models and humans.

## 4. Materials and Methods

### 4.1. Chemicals and Materials

Sephadex G-10 and G-25 gels (medium) were obtained from GE Healthcare (Uppsala, Sweden). 1,1′-Dimethyl-4,4′-bipyridinium dichloride (paraquat), 5-fluoro-2′-deoxyuridine (5-FUdR), 1,1-diphenyl-2-picrylhydrazyl (DPPH) and 2′,7′-dichlorofluorescein diacetate (DCFH-DA) were purchased from Sigma-Aldrich (St. Louis, MO, USA). The TRIzol reagent and RNeasy Mini Kit were obtained from Invitrogen (Carlsbad, CA, USA) and Qiagen (Hilden, Germany), respectively. DNase I, PrimeScript RT and SYBR Premix Ex Taq kits were purchased from Takara (Dalian, China). The bicinchoninic acid (BCA) protein assay kit was purchased from Beyotime (Shanghai, China). Other chemicals used in this study were of analytical grade and obtained from Guangzhou Chemical Reagent Co. (Guangzhou, China). The ultrafiltration centrifuge tube with a molecular weight cut off (MWCO) of 3 kDa was purchased from Millipore (Bedford, MA, USA). The Asiatic hard clam *Meretrix meretrix* was purchased from Huangsha Aquatic Market Co. (Guangzhou, China).

### 4.2. Nematode and Bacterial Strains

The following *C. elegans* strains were used in this study: Bristol N2 (wild-type), GR1352 {*xrIs87 [daf-16(alpha)::GFP::daf-16B + rol-6(su1006)]*} and CF1553 {*muIs84 [(pAD76) sod-3p::GFP + rol-6 (su1006)]*}. *Escherichia coli* strains OP50, NA22 and HT115 were used as food sources for the nematodes as appropriate. All *C. elegan*s and *E. coli* strains were obtained from the Caenorhabditis Genetics Center (University of Minnesota, Minneapolis, MN, USA). The nematodes were maintained under standard conditions at 20 °C unless otherwise stated, and synchronization was performed using the standard alkaline hypochlorite method.

### 4.3. Preparation of Natural Peptides

Fresh *M. meretrix* was used to collect the clam meat, which was mixed with deionized water at a ratio of 1:2 (W/V) and homogenized three times for 15 s at 4 °C on a Polytron homogenizer (CH-6010, Luzern, Switzerland). The homogenate was centrifuged at 6000 × *g* for 30 min at 4 °C, and the supernatant was used for isolation of natural peptides with ethanol as described [49]. After adding ethanol to a final concentration at 60% (*v*/*v*), the mixture was placed at 4 °C for 12 h and centrifuged at 6000 × *g* for 30 min at 4 °C to collect the supernatant. Ethanol was removed from the supernatant by evaporation under reduced pressure in a rotary evaporator (Yarong, Shanghai, China) at 30 °C, and the aqueous solution was freeze-dried with a recovery rate of 9.8% (dry weight). The sample was redissolved in deionized water (10 mg/mL), filtered with a 0.22 μm Millipore filter and desalted by gel filtration chromatography on a Sephadex G-10 column (20 × 1.5 cm). The elution was monitored at 280 nm with a UV detector (Jinda, Shanghai, China), and the tubes containing proteins were combined and freeze-dried as peptide-rich extracts. The protein content of the extract was >70% as determined by the BCA protein assay kit, and the recovery rate from the original sample was 5.7%. The extract was redissolved in deionized water (20 mg/mL) and separated into <3 kDa and >3 kDa fractions using the ultrafiltration tubes with an MWCO of 3 kDa. Antioxidant activity of the fractions was determined by the paraquat resistance assay as described below. The active fractions (0.5 mL, 10 mg/mL) were further separated on a Sephadex G-25 column (150 × 1.2 cm), which was eluted with deionized water at a flow rate of 1 mL/min and monitored at 280 nm with the UV detector. The tubes were pooled according to the UV detection and freeze-dried as *M. meretrix* peptide subfractions for further bioactivity tests.

### 4.4. Paraquat Resistance Assay

Antioxidant activity was tested by the paraquat resistance assay using *C. elegans* as previously described [18]. Briefly, synchronized L1 larvae of N2 nematodes were incubated in S medium containing *E. coli* NA22 at 20 °C for 42 h, and 75 μg/mL of 5-FUdR (final concentration) was added to the young adults. The nematodes were then transferred to 96-well plates (~20 individuals per well, approximately 100 nematodes for each group) containing *E. coli* NA22 as food and peptide samples at the indicated concentrations. After incubation at 20 °C for another 24 h, the nematodes were exposed to 50 mM of paraquat and the survival of nematodes was scored microscopically based on their movement every 12 h until all were dead.

### 4.5. Identification and Synthesis of Peptides

Peptide sequences in the peptide sample were identified by liquid chromatography-tandem mass spectrometry using a reverse-phase nanocolumn (RP-nano-LC-MS/MS) as previously described [18]. Briefly, peptide samples were dissolved in solvent A (water/acetonitrile/formic acid, 98:2:0.1, *v*/*v*/*v*) and loaded onto a ChromXP C18 (3 μm, 120 Å) nanoLC trap column. The desalting procedure was carried out at 2 μL/min for 10 min with 100% solvent A. Then, a linear gradient of 5–35% solvent B (water/acetonitrile/formic acid, 2:98:0.1, *v*/*v*/*v*) over 50 min was used on an analytical column (75 μm × 15 cm C18, 3 μm, 120 Å, ChromXP Eksigent). LC-MS/MS analysis was performed with a TripleTOF 5600 System (AB SCIEX, Concord, Ontario, Canada) fitted with a Nanospray III source (AB SCIEX, Concord, Ontario, Canada). All raw data files (*.wiff) were collectively searched with ProteinPilot Software v. 4.5 (AB SCIEX, Foster City, CA, USA) against a translated protein database of *M. meretrix* nucleotide sequences from the National Center for Biotechnology Information (NCBI). A threshold of confidence above 95% and a local false discovery rate (FDR) of less than 1% were used for peptide identification. Peptides identified in the bioactive peptide subfractions were synthesized by Shanghai Top-peptide Biotechnology Co., Ltd. (Shanghai, China), using the solid-phase synthesis method with a purity of >95%. The synthetic peptides were subjected to antioxidant activity screening using the paraquat resistance assay.

The synthetic peptides with purity >95% were subjected to antioxidant activity screening using the paraquat resistance assay.

### 4.6. Circular Dichroism Spectroscopy

Circular dichroism (CD) spectra of synthetic peptides were obtained and analyzed with a J-815 CD spectrophotometer (Jasco, Tokyo, Japan) as described previously [27]. In brief, the samples were dissolved at a concentration of 0.15 mg/mL in deionized water and phosphate buffered saline (PBS, pH 7.4), respectively, and CD spectra were generated in the far-ultraviolet region from 190 to 240 nm using a quartz cuvette with a path length of 0.1 cm. The DichroWeb online analysis system (http://dichroweb.cryst.bbk.ac.uk) was used to calculate the secondary structure composition through the peptide CD spectra [28].

### 4.7. Nuclear Localization Assay of Transcription Factor DAF-16

The nuclear localization of transcription factor DAF-16 was conducted using transgenic *C. elegans* GR1352 as described [33]. Synchronized GR1352 larvae were first incubated at 20 °C in S medium containing *E. coli* NA22 for 24 h. The nematodes were then treated at 20 °C for 24 h with or without peptide samples. The nematodes were collected and washed three times with a M9 buffer, and then placed on microscope slides coated with 2% agarose, anesthetized with 1% sodium azide and capped with coverslips. A portion of the nematodes treated at 20 °C for 24 h without peptide samples were heat-shocked and used as a positive control. The subcellular localizations of DAF-16::GFP were classified into cytosolic, intermediate and nuclear categories, which were scored in approximately 30 animals per condition with an Olympus BX51 fluorescent microscope (Olympus, Tokyo, Japan). At least 3 independent experiments were performed and data were presented as a ratio of a sub-localization phenotype to the whole population.

### 4.8. Determination of SOD-3 Expression Levels

The transgenic *C. elegan*s CF1553 expressing SOD-3::GFP reporter was used to detect SOD-3 expression [50]. In brief, synchronized CF1553 larvae were first incubated in S medium containing *E. coli* NA22 for 24 h at 20 °C and then treated with peptide samples for another 24 h at 20 °C as above in 24-well plates. To visually observe SOD-3::GFP expression, some of the nematodes were transferred to 384-well plates with 40 μL of M9 buffer containing 1% sodium azide prior to acquiring the fluorescence images with an ImageXpress Micro System (Molecular Devices, Sunnyvale, CA, USA). To determine SOD-3::GFP expression levels, the rest of the nematodes were collected and washed three times with M9 buffer and then homogenized in PBS with 0.1% Tween 20. The lysates were centrifuged at 10,000 × *g* for 5 min at 4 °C and protein content was determined with the BCA protein assay kit as described above. Fluorescence intensity was measured by the Fluoroskan Ascent FL plate reader at an excitation level of 485 nm and an emission level of 530 nm.

### 4.9. Real-time PCR Analysis

Relative quantification of gene expression by real-time PCR was performed essentially as described [39]. Synchronized N2 nematode larvae were incubated in S medium containing *E. coli* NA22 for 24 h at 20 °C and then incubated with peptide samples for another 24 h. The total RNA was extracted from the nematodes with TRIzol reagent according to the manufacturer’s instruction. After reverse transcription, the real-time PCR was performed on a StepOne Plus Real-Time PCR Detection System (Applied Biosystems Inc. Foster, CA, USA) using the SYBR Premix Ex Taq reagent kit. The gene expression levels were analyzed by using the following gene-specific primers: *β-actin* (F: 5′-CCACGAGACTTCTTACAACTCCATC-3′, R: 5′-CTTCATGGTTGATGGGGCAAGAG -3′), *sod-3* (F: 5′-GAGCTGATGGACACTATTAAGCG-3′, R: 5′-GCACAGGTGGCGATCTTCAAG-3′), *ctl-1* (F: 5′-TCTACTCGGATCGTGGAATTCCT-3′, R: 5′-TTGGAACCTTGAGCAGGCTTG-3′), and *ctl-2* (F: 5′-TCCTGTTTTCCGATCGAGGACTC-3′, R: 5′-CTTCACTCCTTGAGTTGGCTTG-3′).

### 4.10. RNA Interference Analysis

RNA interference of *daf-16* was carried out using the feeding method [51]. RNAi plasmids were constructed by cloning a portion of the *daf-16* cDNA into the RNAi vector (pL4440). Primer sequences for the *daf-16* gene amplification are shown as following with the Xba I (TCTAGA) and Kpn I (GGTACC) sites, respectively; daf-16 (F: 5′-TGCTCTAGACTCGATCCGTCACAATCTGTC-3′, R: 5′-CGGGGTACCAGCTGGAGAAACACGAGACGA-3′). The RNAi bacteria *E. coli* HT115 were induced with 1 mM IPTG, collected and added to 96-well plates. Then, synchronized N2 nematode larvae were transferred to the 96-well plates (~20 individuals per well and ~100 nematodes for each group) and incubated for 42 h at 20 °C. After adding 75 μg/mL of 5-FUdR and peptide samples, the plates were incubated for another 24 h. The nematodes were then exposed to 50 mM of paraquat for the oxidative resistance assay as described above.

### 4.11. Statistical Analysis

GraphPad Prism version 7.0 for Microsoft Windows (GraphPad Software, San Diego, CA, USA) was used to generate graphs and perform statistical analysis. *C. elegans* survival and lifespan curves were analyzed by the Kaplan-Meier method and the log-rank test. Probability values of *p* < 0.05 were considered as statistically significant.

## 5. Conclusions

In summary, antioxidant peptides were identified from peptide-rich extract of the Asiatic hard clam *Meretrix meretrix* and were shown to extend the lifespan of *C. elegans*. Further studies indicated that the antioxidant peptides were able to promote nuclear translocation of the transcription factor DAF-16 and to enhance expression of genes in the antioxidant defense system, including *sod-3*, *ctl-1* and *ctl-2*. Using RNAi technology, DAF-16 was found to be required for the peptides to increase the resistance of nematodes to paraquat-induced oxidative stress. Taken together, these results suggest that the antioxidant *M. meretrix* peptides may have dietary health benefits, including stress resistance and age-related disease prevention. Future work is needed to elucidate the absorption and distribution patterns of the peptides in nematodes after feeding and to investigate the effectiveness of the peptides in mouse models and humans.

## Figures and Tables

**Table 1:** Information on HPLC-MS/MS identification and solid-phase synthesis of *M. meretrix* peptides.

Peptide ID	Amino Acid Sequence	Observed m/z	Charge Number	Mascot Score	MW (Da)	Coding Gene	Purity ^a^
MmP1	LCLTPEMVSPTP	644.32	2	56.27	1286.63	gi: 299825528	99.69%
MmP2	THQPCTSPSRLCYLCML	656.96	3	50.21	1967.87	gi: 299827727	99.05%
MmP3	MSPSKLCCPCTLP	690.30	2	51.58	1378.61	gi: 238686243	99.60%
MmP4	LSDRLEETGGASS	661.31	2	60.55	1320.62	gi:299825499	95.37%
MmP5	AGFAGDDAPRAVFPS	739.35	2	82.78	1476.7	gi: 299825528	98.91%
MmP6	GIECLGHYLCHL	679.33	2	53.86	1356.63	gi: 299827928	96.21%
MmP7	YTKFVVESMMP	682.33	2	51.38	1362.62	gi: 299827824	95.39%
MmP8	TLNICVMLVSVNC	712.85	2	52.34	1423.69	gi: 570035797	96.65%
MmP9	KSYGCEKGTGCLLL	736.36	2	60.21	1470.72	gi: 299825528	97.13%
MmP10	QPCTLPSRPCSLCT	753.35	2	54.9	1504.68	gi: 299825528	98.95%
MmP11	KEGCREPETEKGHR	828.41	2	61.14	1654.78	gi: 299825499	96.51%
MmP12	LSDRLEETGGASSIQHE	610.29	3	54.51	1827.86	gi: 299825499	95.45%
MmP13	RPVCERNTRKPSMTC	889.43	2	71.39	1776.85	gi: 299825499	99.90%
MmP14	GGEINCRIN	488.24	2	55.59	974.46	gi: 299827820	97.75%
MmP15	ANIHGWCVKGFEHDL	440.22	4	57.89	1756.8	gi: 299827878	98.57%
MmP16	GFAGDDAPRAVFPSIVG	838.42	2	67.3	1674.84	gi: 299825528	99.06%
MmP17	EYEINCRINCR	706.83	2	52.92	1411.63	gi: 299825354	98.11%
MmP18	VSYRAETCHALL	681.85	2	59.75	1361.68	gi: 570035646	99.06%
MmP19	IVTNWDDMEK	625.79	2	77.36	1249.56	gi:299827878	97.21%
MmP20	LPMVITCP	437.23	2	55.68	872.45	gi:299827878	96.39%
MmP21	LLTMAPVCA	459.74	2	66.76	917.47	gi: 299827727	97.56%
MmP22	CRNSVSRTAAPCL	689.33	2	63.83	1376.67	gi: 238685652	97.46%
MmP23	LGECLLCPG	452.72	2	61.57	903.42	gi: 299825408	99.44%
MmP24	MATYMAGVLKVSNM	774.38	2	52.99	1546.72	gi: 299827727	98.38%
MmP25	VECWGIECLGH	639.26	2	56.98	1276.52	gi: 299827928	98.87%

^a^ Purity of peptides synthesized.
